# AQbD-assisted HPLC method for simultaneous determination of methylparaben and propylparaben in liquid oral dosage forms

**DOI:** 10.1038/s41598-026-54751-2

**Published:** 2026-05-29

**Authors:** Raneem Darraj, Mohammad Haroun, Ayat abbood, Ibrahim alghoraibi

**Affiliations:** 1 Department of Pharmaceutical Chemistry and Quality Control of Drugs, Faculty of Pharmacy, Latakia University, Latakia, Syria; 2 Department of Physics, Faculty of Science, Damascus University, Damascus, Syria

**Keywords:** Analytical quality by design (AQbD), HPLC, Methylparaben, Propylparaben, Pharmaceutical syrups, Chemistry, Drug discovery

## Abstract

The determination of preservatives like methylparaben (MP) and propylparaben (PP) in pharmaceutical syrups is critical for quality control. Traditional one-factor-at-a-time (OFAT) method development often fails to account for interactions between variables, leading to less robust procedures. This study aims to develop and validate a robust HPLC method for the simultaneous determination of MP and PP using a systematic Analytical Quality by Design (AQbD) approach. An initial risk assessment (Ishikawa diagram and Failure Mode and Effects Analysis) identified five critical method attributes (CMAs): mobile phase type, organic phase percentage, pH, flow rate, and column temperature. These CMAs were systematically optimized using a D-optimal design (34 runs) with resolution, retention time, and tailing factor defined as critical quality attributes (CQAs). Statistical analysis revealed that organic solvent type and percentage were the most significant factors affecting resolution. The Method Operable Design Region (MODR) was established, and the optimal conditions were predicted as 40 °C, 1.5 mL/min flow rate, pH 6.1, and 67.7% methanol, achieving baseline separation within 6 min. The method was validated per ICH Q2(R2) guidelines, demonstrating excellent linearity (R² > 0.99 for both analytes), precision (%RSD < 2%), and accuracy (recovery 98.2–99.8%). The method was successfully applied to quantify MP and PP in commercial pharmaceutical syrups, with results falling within expected ranges. The AQbD approach ensured a robust, well-understood, and reliable method, suitable for routine quality control and capable of withstanding minor variations in chromatographic conditions.

## Introduction

Determination of preservatives in pharmaceutical formulations is a critical aspect of quality control, particularly for parabens such as methylparaben (MP) and propylparaben (PP) in oral syrups^1^. Accurate control of paraben concentration is essential for both product stability and patient safety^2^. This importance has grown due to increasing reports highlighting the presence of parabens in pharmaceutical products and ongoing studies investigating potential links to health concerns, including potential endocrine-disrupting effects^3^. Consequently, there is a rising demand for improved analytical methods to ensure their accurate quantification. Several analytical methods for paraben determination have been reported in the scientific literature, including gas chromatography (GC)^4^ electrochemical analysis^5^ and UV-spectroscopy^6^. Each technique offers distinct advantages: GC provides high sensitivity^4^, electrochemical methods enable rapid detection^5^ and UV-spectroscopy offers simplicity and cost-effectiveness^6^. However, high-performance liquid chromatography (HPLC)^7^ remains the most reliable methodology for routine quality control due to its superior resolution, high sensitivity, excellent precision, and elimination of derivatization requirements typically needed in GC analysis^8^. HPLC’s versatility allows simultaneous determination of multiple parabens in complex matrices while maintaining chromatographic efficiency^8^. Traditional development approaches for analytical methods often depend on one-factor-at-a-time (OFAT) optimization, where each parameter is developed independently without studying interactions between variables^9^. This linear approach fails to capture synergistic or antagonistic effects among critical method parameters such as mobile phase composition, pH, flow rate, and column temperature. Consequently, methods developed through OFAT frequently lack robustness, exhibit narrow operable ranges, and require extensive re-optimization when transferred between instruments or laboratories^9^. These limitations present significant challenges for pharmaceutical quality control, which demands consistent, transferable analytical procedures. To address these limitations, the Analytical Quality by Design (AQbD) approach has emerged as a systematic framework that enhances method robustness while aligning with regulatory guidelines outlined in ICH Q8-Q11. The AQbD methodology employs a structured workflow beginning with definition of an Analytical Target Profile (ATP)^10^, followed by systematic risk assessment to identify Critical Method Attributes (CMAs) and Critical Quality Attributes (CQAs). Through Design of Experiments (DOE), multivariate relationships are explored, leading to establishment of a Method Operable Design Region (MODR) where method performance is assured despite minor parameter variations^11^. This science-based approach facilitates knowledge-driven development, reduces method failure risks, and supports regulatory submissions through enhanced understanding of method capabilities and limitations^12^. Despite its demonstrated benefits in pharmaceutical analysis, comprehensive AQbD application to preservative analysis in syrup formulations remains inadequately explored. Previous investigations have exhibited three primary limitations such as partial implementation of AQbD principles, often focusing solely on DOE without complete workflow integration^13^, application to simpler matrices like cosmetic products rather than complex pharmaceutical syrups containing multiple excipients^14^, and lack of practical demonstration regarding MODR utility in routine quality control settings^15^. Pharmaceutical syrups present unique analytical challenges including high viscosity, sugar content, and potential interference from flavors and colors, necessitating specifically tailored analytical approaches that account for these matrix effects^16^.

Several HPLC methods have been reported for the simultaneous determination of methylparaben (MP) and propylparaben (PP) in pharmaceutical formulations. However, the novelty of the present work lies not in the analytes themselves but in the systematic application of Analytical Quality by Design (AQbD) principles. To the best of our knowledge, a complete AQbD framework—comprising explicit definition of the Analytical Target Profile (ATP), quantitative Failure Mode and Effects Analysis (FMEA) for risk assessment, D-optimal design for multivariate optimization, and experimental verification of the Method Operable Design Region (MODR)—has not been previously applied to the simultaneous determination of MP and PP in syrups. The FMEA step is particularly critical, as it quantitatively identifies and prioritizes critical method attributes (CMAs) before DoE, ensuring that the experimental design focuses on truly influential factors (RPN ≥ 140) while excluding irrelevant or low-risk parameters. This risk-based approach improves experimental efficiency, reduces model uncertainty, and is a defining feature that distinguishes AQbD from conventional DoE-only optimization.

Therefore, this study aims to develop and validate a robust HPLC method for the simultaneous determination of MP and PP in pharmaceutical syrups using a systematic Analytical Quality by Design (AQbD) approach. Specific objectives include: (i) implementing a complete AQbD workflow from Analytical Target Profile (ATP) definition through Method Operable Design Region (MODR) establishment; (ii) employing risk assessment (FMEA) and multivariate experimental design (D-optimal design) to optimize critical chromatographic conditions; (iii) validating the optimized method according to ICH Q2(R2) guidelines^17^; and (iv) demonstrating practical applicability through analysis of commercial syrup products from three different manufacturers. This work contributes to pharmaceutical quality control by providing a systematically developed method with a defined design space, which enhances method robustness and facilitates future method transfer.

## Experimental

### Chemical and reagents

The analytical reference standards of MP (≥ 98% purity) and PP (≥ 98% purity) were procured from SRL Pvt. Ltd., India. Potassium dihydrogen phosphate (KH_2_PO_4_ ≥99% purity) was obtained from SRL Pvt. Orthophosphoric acid (85% w/w, analytical grade) and sodium hydroxide (NaOH, ≥ 98% purity) were purchased from Chem-Lab NV (Belgium) and SRL Pvt. Ltd. (India), respectively. HPLC-grade acetonitrile and methanol (99.9%) were purchased from Sigma-Aldrich, Belgium. Distilled water was supplied by a local pharmaceutical company, Sterile syringe filter unit equipped with a 0.45 μm hydrophilic PTFE membrane. All pharmaceutical syrup samples were purchased from local pharmacies.

### Apparatus

The analysis was conducted on a Shimadzu LC-2030 C.HPLC system (Japan) comprising a solvent delivery pump, an autosampler, a column oven, and a UV detector. Chromatographic separation was carried out using a Kromasil^®^ C18 column (150 mm × 4.0 mm internal diameter, 5 μm particle size) with a pore size of 100 Å and a carbon load of 20%. The column was protected by a guard column of the same stationary phase (10 mm × 4.0 mm, 5 μm). Column chemistry and silanol activity were not characterized, as a commercial column was used according to standard practice. Specific conditions tested during the optimization process are summarized in Table 2. System control and data processing were handled by the LabSolution software. Various laboratory equipment included: volumetric flasks and pipettes, ultrasonic bath (Sartorius), pH meter (Sartorius), analytical balance, magnetic stirrer (Jaken digital).

Column conditioning and temperature equilibration: Prior to each sequence, the column was conditioned by passing the mobile phase at the specified flow rate for 30 min. After setting the column temperature to the desired value (30–40 °C depending on the experimental run), the system was allowed to equilibrate for 30 min with mobile phase flowing at the specified flow rate before the first injection. Between different mobile phase compositions (e.g., during DoE runs), a wash step with 100% organic solvent (methanol or acetonitrile) for 15 min was performed, followed by re-equilibration with the new mobile phase for 20 min. For routine QC applications, the column is stored in 70% methanol: water between runs. Before each daily analysis, the column is re-equilibrated for 20 min with the optimized mobile phase (67.7% methanol: phosphate buffer, pH 6.1) at 1.5 mL/min and 40 °C.

### Preparation of mobile phases and standard solutions

#### Mobile phases

To determine the optimal conditions for paraben separation, a series of mobile phases were prepared and tested. These mobile phases consisted of binary mixtures prepared with two different organic modifiers: water-acetonitrile and water-methanol separately at organic solvent concentrations of 60%, 70%, and 80% (v/v). The pH of these mixtures was adjusted to 4.5, 5.5, and 6.5 using a phosphate buffer. The pH of the buffer solution was adjusted to the desired value using orthophosphoric acid or 0.1 M sodium hydroxide solution before mixing with the organic modifier. Then, the mobile phases passed through 0.45 μm filter and degassed for 15 min in ultrasonic bath.

#### Standard solutions

Standard solutions were prepared in the same solvent as the mobile phase for each experimental run (methanol for methanol-based mobile phases, acetonitrile for acetonitrile-based mobile phases) to ensure sample-mobile phase compatibility and avoid solvent-induced artifacts (e.g., peak splitting or retention time shifts). This is essential for the D-optimal design, where mobile phase type (methanol vs. acetonitrile) is included as a categorical factor.

Two separate stock solutions were prepared using the same procedure but with different mobile phases. The first was prepared using (methanol: water), and the second was prepared using (acetonitrile: water). For each, 100 mg of MP and 20 mg of PP reference standards were accurately weighed into a 100 mL volumetric flask, dissolved in approximately 70 mL of the corresponding solvent, sonicated for 10 min to ensure complete dissolution, and then diluted to volume with the same solvent. This yielded primary stock solutions containing 1000 µg/mL of MP and 200 µg/mL of PP in methanol and, separately, in acetonitrile. For the Design of Experiments (DoE) study, working standard solutions were prepared daily by diluting the appropriate stock solution in the mobile phase composition corresponding to that specific experimental run. For method validation, calibration standards for linearity were prepared over the concentration range of 50–250 µg/mL for MP and 10–50 µg/mL for PP by appropriate dilution of the stock solution with the optimized mobile phase.

### Sample preparation

Three different commercial pharmaceutical syrup products, each containing both methylparaben (MP) and propylparaben (PP) as preservatives, were purchased from local pharmacies. These syrups represented a variety of therapeutic categories, including antipyretics, decongestants, antitussives, and non-steroidal anti-inflammatory drugs (NSAIDs). The products originated from three different manufacturers (coded as Company A, Company B, and Company C for confidentiality). The exact concentrations of MP and PP were not declared on the product labels. For the purpose of this study, five samples were analyzed in total, consisting of: two different batches from Company A, two from Company B, and one from Company C. All samples were within their stated expiry dates at the time of analysis. Based on the optimized conditions obtained from the AQbD approach, the mobile phase consisted of methanol and phosphate buffer (pH 6.1) in a ratio of 67.7:32.7 (v/v). The flow rate was set at 1.5 mL/min, column temperature at 40 °C, and detection wavelength at 254 nm. This wavelength is the well-established λmax for the simultaneous detection of MP and PP in reversed-phase HPLC systems, as consistently documented in the analytical literature. Sample preparation was performed by diluting 1.0 mL of syrup to 10 mL with the mobile phase, followed by sonication for 5 min and filtration through a 0.45 μm PTFE filter.

Filtration was performed using a 0.45 μm PTFE syringe filter. PTFE membranes were selected for their excellent chemical compatibility with methanol-water and acetonitrile-water mobile phases. To verify the absence of analyte adsorption, an adsorption test was performed: a standard solution containing MP (100 µg/mL) and PP (20 µg/mL) was filtered through the PTFE membrane, and the filtrate was compared to an unfiltered standard. Recoveries were 99.2% for MP and 98.7% for PP, confirming negligible adsorption (< 2%).

### HPLC method development by AQbD approach

#### Selection of analytical target profile (ATP), critical quality attributes (CQAs) and critical method attributes (CMAs)

The Analytical Target Profile (ATP) was explicitly defined prior to method development as follows: a reversed-phase HPLC method capable of simultaneously determining methylparaben (MP) in the range of 50–250 µg/mL and propylparaben (PP) in the range of 10–50 µg/mL in pharmaceutical syrups. The method must meet the following quantitative acceptance criteria:

Resolution (Rs) between MP and PP peaks ≥ 2.0.

Tailing factor (Tf) ≤ 1.5 for both analytes.

Retention time of the last eluting peak ≤ 10 min.

Precision (%RSD) ≤ 2.0% for both intra-day and inter-day.

Accuracy (recovery) between 98 and 102%.

Limit of detection (LOD) ≤ 0.5 µg/mL for each analyte.

Limit of quantification (LOQ) ≤ 2.0 µg/mL for each analyte.

These criteria were derived from ICH Q2(R2) guidelines and typical quality control requirements for preservative analysis in liquid oral formulations^18^. CQAs are the measurable output responses of the method that are critical to its performance. In HPLC, these include resolution (Rs ˃ 2), tailing factor (T_f_ ˂ 1.5) and retention time.

CMAs are the physical, chemical, or biological properties of the input materials that can affect the performance of the method. For the simultaneous determination of MP and PP, the following CMAs were identified based on molecule-specific considerations:

- Mobile phase pH: MP and PP are weak acids with pKa values of approximately 8.2–8.5 (MP) and 8.3–8.5 (PP). Within the studied pH range (4.5–6.5), both analytes remain in their non-ionized form, maximizing retention on the C18 stationary phase and avoiding peak tailing due to interactions with residual silanol groups.
- Organic solvent type: Methanol (protic) provides hydrogen bonding interactions with the hydroxyl group of parabens, enhancing selectivity between MP and PP, whereas acetonitrile (aprotic) offers higher elution strength but lower resolution.
- Organic solvent percentage: The percentage of organic modifier directly controls elution strength. Higher percentages reduce retention times for both analytes but may compromise resolution, especially for PP, which is more hydrophobic.
- Flow rate: The similar molecular weights of MP (152.15 g/mol) and PP (180.20 g/mol) require efficient separation. Flow rate affects column efficiency via the van Deemter equation and was optimized to balance resolution and analysis time.
- Temperature: Temperature affects mobile phase viscosity and analyte diffusion. PP, being more hydrophobic and larger than MP, shows greater sensitivity to temperature changes, as confirmed by our DoE results (*p* = 0.02 for PP retention time).

These factors directly affect the CQAs (resolution, retention time, and tailing factor) and were systematically investigated using D-optimal design^19^.

#### Risk assessment

**Fig. 1:**
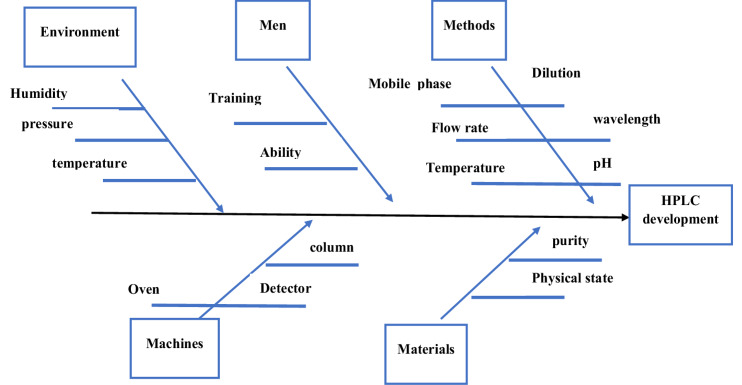
Ishikawa (fish-bone diagram) to identify potential risks for HPLC development.

An important part of AQbD approach is risk assessment as set in ICH Q8 and ICH Q9 guidelines. To visualize and categorize these potential risks, analytical scientists employ various established tools. Initial hazard identification is often facilitated by constructing cause-and-effect diagrams, commonly known as Ishikawa or fishbone diagrams^20^ (Fig. 1), which help map out all possible variables affecting the method. Following identification, risks are evaluated and prioritized to focus development efforts on the most critical factors. This prioritization is frequently achieved through a Failure Mode and Effects Analysis (FMEA)^21^. FMEA is a systematic, proactive method for evaluating a process to identify where and how it might fail and to assess the relative impact of different failures, allowing the team to categorize risks into different levels (e.g., high, medium, low) based on their severity, occurrence, and detectability.

To systematically identify and prioritize Critical Method Attributes (CMAs) for the Design of Experiments, a quantitative Failure Mode and Effects Analysis (FMEA) was performed. Each potential parameter was evaluated using three risk components: Severity (S, impact on method performance), Occurrence (O, probability of failure), and Detectability (D, ease of detecting failure). The Risk Priority Number (RPN) was calculated as S × O × D. Based on the FMEA results (Table 1), five CMAs with RPN ≥ 140 (mobile phase composition, mobile phase pH, flow rate, temperature, and mobile phase type) were classified as high-risk and selected for systematic investigation using a D-optimal design. Although wavelength (RPN = 120), injection volume (RPN = 120), column type (RPN = 84), and sample dilution (RPN = 84) showed medium risk, they were excluded for the following justifications: wavelength was fixed at 254 nm, the established λmax for both MP and PP based on UV spectra and literature; injection volume was fixed at 20 µL based on preliminary linearity tests demonstrating no peak distortion or column overloading up to 30 µL; column type was kept constant (Kromasil C18) as a commonly available stationary phase with adequate retention and resolution in preliminary trials; and sample dilution (1:10 with mobile phase) was standardized based on expected concentration ranges to ensure compatibility with the mobile phase and prevent viscosity issues. This risk-based approach ensured that development efforts focused on high-risk parameters while medium-risk factors were controlled through fixation, in accordance with AQbD principles.

**Table 1:** Failure Mode and Effects Analysis (FMEA) for HPLC development.

Parameter	Effect	Severity (s)	Probability to occur (O)	Detectability (D)	RPN	Risk level
Mobile phase composition	Change in retention time, resolution and shape	8	5	6	**240**	**High**
Mobile phase Ph	Change in retention time, resolution and shape	8	5	6	**240**	**High**
Flow rate	Change in retention time and resolution	8	4	6	**192**	**High**
Temperature	Resolution	7	4	5	**140**	**High**
Wavelength	Selectivity and sensitivity	6	4	5	120 (medium risk)	Medium
Column type	Change in retention time and resolution	7	4	3	84 (medium risk)	Medium
Injection volume	Peak shape and overloading of column	6	4	5	120 (medium risk)	Medium
Sample dilution	Peak shape and incompatibility with mobile phase	7	3	4	84 (medium risk)	Medium

Risk Priority Number (RPN) = S*O*D.

Factors in Bold: selected for design of experiment as CMAs.

#### Design of experiments (DoE)

To systematically optimize the chromatographic conditions, a Design of Experiments (DoE) approach based on Quality by Design principles was employed. Based on the risk assessment outcomes, five factors were identified as Critical Method Attributes (CMAs) requiring systematic investigation. These include mobile phase type (categorical: methanol vs. acetonitrile), mobile phase pH, organic solvent percentage, flow rate, and column temperature. The selected responses, defined as Critical Quality Attributes (CQAs), are resolution between methylparaben (MP) and propylparaben (PP) peaks, tailing factor for each paraben, and retention time of the last eluting peak (Table 2).

A D-optimal design was chosen due to its flexibility in handling mixed factor types (continuous and categorical) and its efficiency in estimating main effects, interactions, and quadratic terms. The D-optimal design was selected over other designs (e.g., central composite design or Box-Behnken) for the following reasons: (i) it efficiently handles mixed factor types including categorical factors (mobile phase type: methanol vs. acetonitrile), which standard response surface designs cannot accommodate; (ii) it allows for an irregular design space with asymmetric factor ranges; (iii) it provides flexibility to add center points and replicates for lack-of-fit testing; and (iv) it minimizes the number of experimental runs while maintaining adequate power to estimate main effects, two-way interactions, and quadratic terms^22^. The D-efficiency of the generated design was 0.78, indicating good design efficiency^23^. Unlike regular fractional factorial designs, D-optimal designs do not possess a traditional ‘design resolution’ value. Instead, design quality was assessed using D-efficiency (0.78) and variance inflation factors (VIFs), all of which were < 5, indicating no significant aliasing among main effects^24^. A total of 34 experimental runs (including 5 center points and 3 replicates) was generated. This number is statistically justified for a 5-factor mixed-design D-optimal model (one categorical factor: mobile phase type; four continuous factors: temperature, flow rate, pH, and organic phase percentage). D-optimal designs are not required to be balanced or orthogonal, and 34 runs represent an efficient balance between model estimation power (main effects, two-way interactions, and quadratic terms) and experimental economy^22^. For comparison, a full factorial design with the same factors would require 2^4^ × 2 = 32 runs for continuous factors alone, plus additional runs for the categorical factor and replicates. Therefore, 34 runs are appropriate and not excessive for this complexity. The design matrix was generated using Design-Expert^®^ software (Version 13, Stat-Ease Inc.), and the total number of experimental runs was determined based on the model requirements. All experiments were performed in random order to minimize the impact of uncontrolled variables^23^, and the obtained data were used to establish the Method Operable Design Region (MODR) where all CQAs consistently meet predefined acceptance criteria^17^.

Model validation was performed using a combination of internal diagnostics (residual plots, lack-of-fit tests) and external experimental verification^23^. Nine independent validation runs were performed at MODR boundaries (Table 4) to confirm model predictions^17^. External test set validation (independent runs not used in model building) is recommended for future work but was beyond the scope of this study.

**Table 2:** Critical Method Attributes (CMAs), Critical Quality Attributes (CQAs), and their studied ranges investigated in the D-optimal design.

Factor	Name	Units	Type	Sub type	Minimum	Maximum	CQAs
A	Temperature	C^˚^	Numeric	Continuous	30	40	-Resolution (R_s_) -Retention time (R_t)_ -Tailing factor (T_f_)
B	Flow rate	mL/min	Numeric	Continuous	0.8	1.5	
C	pH		Numeric	Continuous	4.5	6.5	
D	Percent of mobile phase	%	Numeric	Continuous	60	80	
E	Mobile phase		Categoric	Nominal	Methanol	Acetonitrile	

#### Optimization of chromatographic conditions

By employ optimization process, MODR is obtained. MODR is the design space in which the analytical method is expected to meet the ATP. It helps to identify and optimize CMAs where all CQAs remain within their acceptance criteria despite minor variations. Using the reduced quadratic regression models generated from the 34 experimental runs, contour plots were constructed for each response, and an overlay plot was created to identify the region where all CQAs were simultaneously satisfied.

#### Method validation protocol

An internal standard was not used in this method. This decision was based on the observation that the optimized conditions yielded excellent precision (%RSD < 1.5% for both intra-day and inter-day studies) and accuracy (recovery 98–102%) without an internal standard, as permitted by ICH Q2(R2) guidelines when method performance is adequate. For routine quality control applications where matrix complexity is low (e.g., syrups with known excipients), the omission of an internal standard is acceptable and simplifies the analytical workflow. However, for more complex matrices or when higher quantitative certainty is required, the inclusion of an internal standard (e.g., ethyl paraben) is recommended.

The optimized chromatographic conditions were validated according to ICH Q2 (R2) guidelines. System suitability, linearity, accuracy, precision, limit of detection (LOD) and limit of quantification (LOQ) were performed using the final optimized condition selected from within MODR to ensure the method suitability for routine quality control of MP and PP in pharmaceutical syrups.

#### System suitability

A system suitability test was performed by the injection of six (*n* = 6) replicates of a standard working solution containing MP (100 µg/mL) and PP (20 µg/mL) under the optimized chromatographic conditions. The solution was injected using a fixed injection volume of 20 µL.

#### Linearity

Linearity was assessed by analyzing five calibration standards across the concentration ranges of 50–250 µg/mL for methylparaben (MP) and 10–50 µg/mL for propylparaben (PP). The optimized conditions eliminated the need for an internal standard. Calibration curves were constructed by plotting peak areas against nominal concentrations, and the data were fitted by least‑squares linear regression. The correlation coefficients (R^2^) and regression equations were calculated for both analytes.

#### Precision

intra‑day precision was determined by analyzing three concentration levels (80,100,120)% of standard solutions in triplicate (*n* = 3) of a standard mixture containing 100 µg/mL MP and 20 µg/mL PP. The relative standard deviation (%RSD) was computed.

Inter-day precision was assessed by analyzing the previous solution on a different day. The overall %RSD across the two days was determined to evaluate day‑to‑day variability.

#### Accuracy

Accuracy was demonstrated through a recovery study. The recovery study was performed using a representative commercial syrup sample (Sample 1, Company A, which contained approximately 100 µg/mL MP and 20 µg/mL PP as determined by preliminary analysis). The native paraben content of the syrup was first quantified. Known amounts of MP and PP standards were then spiked into the syrup to achieve three concentration levels corresponding to 50%, 100%, and 150% of the baseline concentration. Each level was prepared and analyzed in triplicate. The percentage recovery and its %RSD were calculated for each analyte at every level.

To specifically address potential interference from excipients, a placebo solution containing all syrup excipients (sucrose, glycerin, flavors, and colors) at their maximum labeled concentrations was analyzed.

#### Robustness

Robustness was established according to the AQbD paradigm through the definition and experimental verification of the Method Operable Design Region (MODR). The MODR boundaries were experimentally challenged at their extreme corners and at worst-case combinations (as presented in Table 3, Results section). Within the MODR, all Critical Quality Attributes (CQAs) were confirmed to meet acceptance criteria despite deliberate variations in Critical Method Attributes (CMAs), including temperature (± 2 °C), flow rate (± 0.1 mL/min), pH (± 0.2), and organic phase percentage (± 2.5%).

#### Limit of detection (LOD) and limit of quantification (LOQ)

LOD and LOQ were calculated from the calibration curves using the standard deviation of the intercept (σ) and the mean slope (S) according to the formulas: LOD = 3.3 σ/S and LOQ = 10 σ/S. The obtained values confirmed the method’s sensitivity for the intended application.

## Results and discussion

### Risk assessment

The Ishikawa diagram (Fig. 1) identified potential CMAs affecting chromatographic performance. Quantitative FMEA (Table 1) ranked the risk of each parameter, identifying mobile phase composition, pH, and type as high-risk (RPN ≥ 140), while flow rate and temperature (RPN = 192 and 140, respectively) were also included for comprehensive method understanding. Medium-risk factors (wavelength, injection volume, column type, sample dilution; RPN 84–120) were excluded with justifications: wavelength fixed at 254 nm (λmax of both parabens), injection volume fixed at 20 µL (linearity confirmed up to 30 µL), column type fixed as Kromasil C18 (adequate preliminary performance), and dilution fixed at 1:10 (compatibility with mobile phase). This systematic, risk-based selection of CMAs addresses the limitations of arbitrary factor selection and ensures that DoE efforts focus on truly critical parameters.

### Optimization chromatographic conditions and MODR establishment

A D-optimal design was employed to investigate the five selected CMAs. The 34 experimental runs (Table 3) were performed, and the responses (Rs, T_f_, and R_t_) were fitted to quadratic polynomial models. The chromatographic results revealed that all investigated factors significantly influence the separation performance of methyl and propyl parabens. Increasing temperature, flow rate, or organic phase ratio consistently reduces retention times but potentially compromising resolution. pH exhibited a marked effect on peak symmetry (tailing factor). The organic solvent type plays a critical role: acetonitrile tends to provide shorter retention times and, whereas methanol offers higher resolution. The observed interactions among factors, such as the temperature effect varying with solvent type, underscore the necessity of statistical optimization to achieve a balanced compromise between speed, resolution, and peak shape.

ANOVA analysis was performed for each variable followed by 3D surface of model graph.

**Table 3:** Experimental results of responses in method development.

Run	Independent factors											Dependent factors										
	A			B		C		D			E	*R*_s_			*R*_t_ (MP)		*R*_t_((PP)		T_f_(MP)		T_f_(PP)	
1	40			1.5		4.5		80			A	1.771			2.072		2.403		1.554		1.562	
2	40			1.227		6.49		67.7			M	6.885			3.618		3.788		1.565		1.513	
3	40			0.8		4.5		60			A	5.569			4.834		6.886		1.776		1.685	
4	35			1.15		5.5		70			A	3.158			2.962		3.758		1.444		1.472	
5	30			1.5		4.5		80			M	2.927			2.502		3.204		1.566		1.648	
6	30			1.5		4.5		80			M	3.127			2.303		2.978		1.642		1.723	
7	30.55			1.115		5.29		60			A	5.778			3.622		5.269		1.452		1.483	
8	35			1.15		5.5		70			A	2.645			1.657		2.122		1.78		1.89	
9	35			1.15		5.5		70			M	6.27			2.959		4.605		1.445		1.375	
10	36.3			0.8		4.68		65.8			M	8.584			5.937		10.255		1.574		1.517	
11	35			1.15		5.5		70			M	6.272			2.955		4.612		1.442		1.377	
12	35			1.15		5.5		70			M	7.867			3.694		5.386		1.676		1.769	
13	35			1.15		5.5		70			A	4.756			4.522		5.967		1.667		1.786	
14	30			1.5		6.5		72.2			A	3.106			2.311		2.951		1.411		1.431	
15	30			1.5		4.5		60			A	5.389			2.687		3.879		1.413		1.432	
16	40			1.5		4.5		60			M	13.117			4.146		9.153		1.463		1.449	
17	30			0.8		6.3		60			A	5.583			5.036		7.334		1.482		1.548	
18	40			1.5		6.5		80			M	2.021			2.398		2.991		1.51		1.493	
19	33.2			1.5		5.62		80			A	1.84			2.092		2.44		1.399		1.397	
20	40			0.8		4.5		60			A	5.569			4.834		6.886		1.472		1.492	
21	40			0.8		5.81		80			A	1.974			3.851		4.466		1.439		1.438	
22	30			0.821		5.67754		67.6212			M	7.694			5.816		9.736		1.568		1.53	
23	30			1.5		6.5		60			M	13.304			5.978		12.912		1.538		1.409	
24	40			0.8		6.5		60			M	12.786			6.792		13.525		1.41		1.237	
25	30			0.8		4.5		60			M	11.778			7.549		16.207		1.785		1.849	
26	30			0.8		6.5		80			M	3.129			4.586		5.854		1.564		1.565	
27	36			0.8		6.5		70.4			A	3.268			4.236		5.366		1.488		1.506	
28	34.6			1.15		6.5		80			A	1.912			2.707		3.147		1.396			1.396
29	36			0.8		4.5		80			M	3.045			4.505		5.688		1.556			1.546
30	40			1.5		6.5		60			A	5.042			2.604		3.729		1.489			1.539
31	36.75			1.5		5.25		61			A	5.334			2.623		3.768		1.418			1.435
32	30			0.8		4.5		80			A	2.076			3.91		4.588		1.422			1.432
33	30			1.5		6.5		60			M	13.304			5.977		12.897		1.4987			1.397
34	40			1.08		4.5		80			M	2.831			3.305		4.136		1.549			1.534

*A= Temperature (^˚^C), B= Flow rate (mL/min), C = pH, D= Percent of organic phase (%), E= Type of organic phase (M: methanol (minimum), A: acetonitrile (maximum)).

*R_s_: resolution, R_t_ retention time, T_f_: tailing factor.

### DoE: statistical response analysis

#### The effect of factors on resolution

A quadratic equation describing the individual main effects on resolution was generated by reduced quadratic model, as presented in Eq. (1):

1 $${\text{Resolution }}\left( {{\mathrm{Rs}}} \right) = {\text{ 4}}.{\mathrm{96125}} - {\mathrm{3}}.{\mathrm{35}}0{\text{24 D}} - {\mathrm{1}}.{\text{8847 E }} + {\text{ 1}}.{\text{58713 DE }} + {\text{ }}0.{\mathrm{8}}0{\text{751 D}}^{{\mathrm{2}}}$$

The ANOVA results for effect of factors on resolution showed significant model (p-value ˂ 0.05) with a high F-value and a non-significant lack of fit (*p* > 0.05), indicating good model predictability (R² = 0.975, Adjusted R^2^ =0.972 Predicted R² = 0.967). The model factors: organic phase concentration (D), solvent type (E) and their interaction (DE) and D^2^ were significant (p-value ˂ 0.05). This indicate that improving the resolution between MP and PP will primarily be achieved by fine tuning the solvent type and its percentage. This can be explained by Acetonitrile (aprotic) has higher elution strength, reducing retention times; methanol (protic) enhances selectivity via hydrogen bonding, improving resolution. Other main effects (A, B,C) and their interaction were not mentioned significant.

**Fig. 2:**
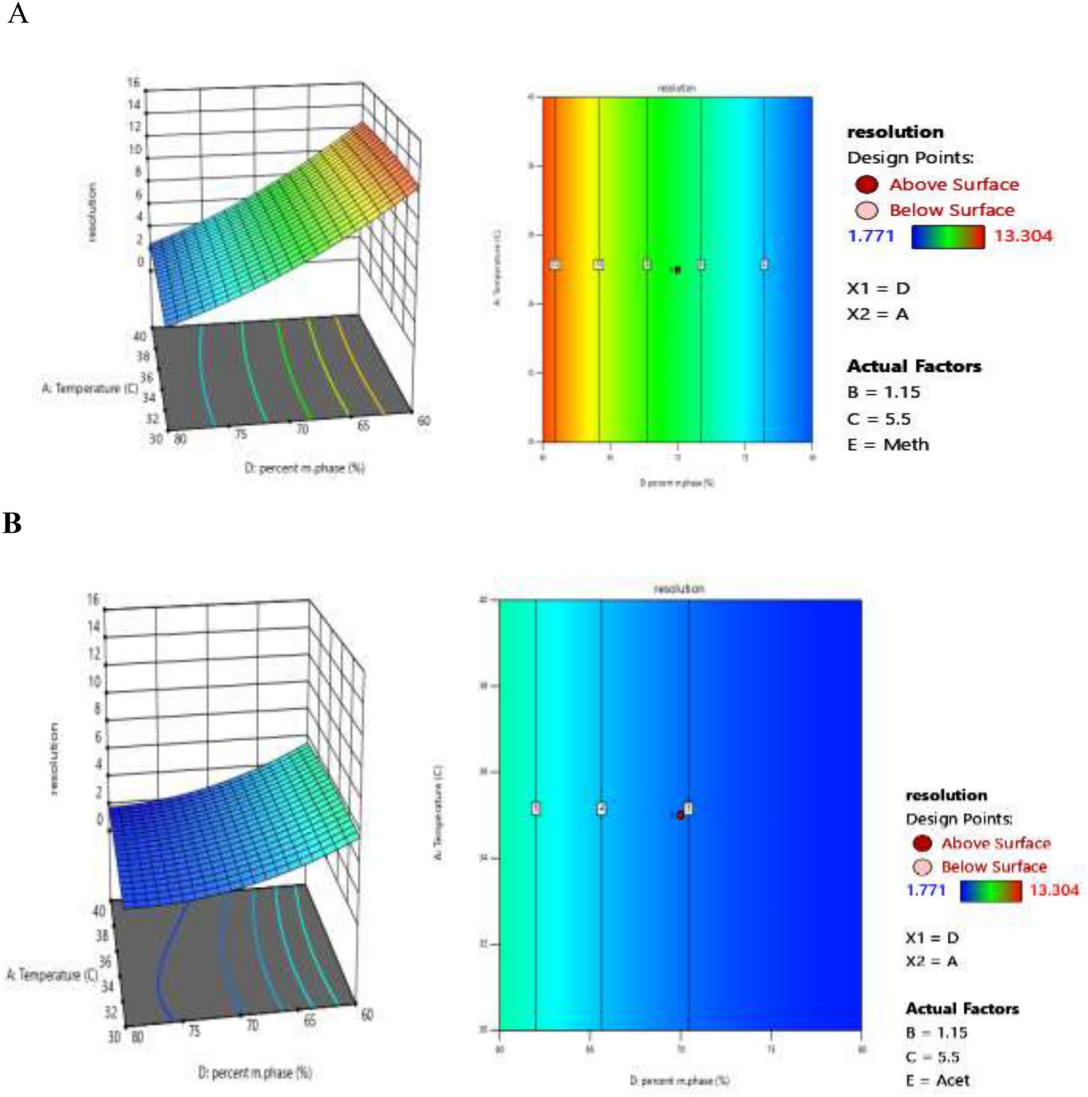
Contour plots (2D) and response surface plots (3D) showing the combined effects of organic phase percentage (X₁) and temperature (X₂) on chromatographic resolution. Results are presented for two organic solvents: (**A**) methanol and (**B**) acetonitrile.

**Fig. 3:**
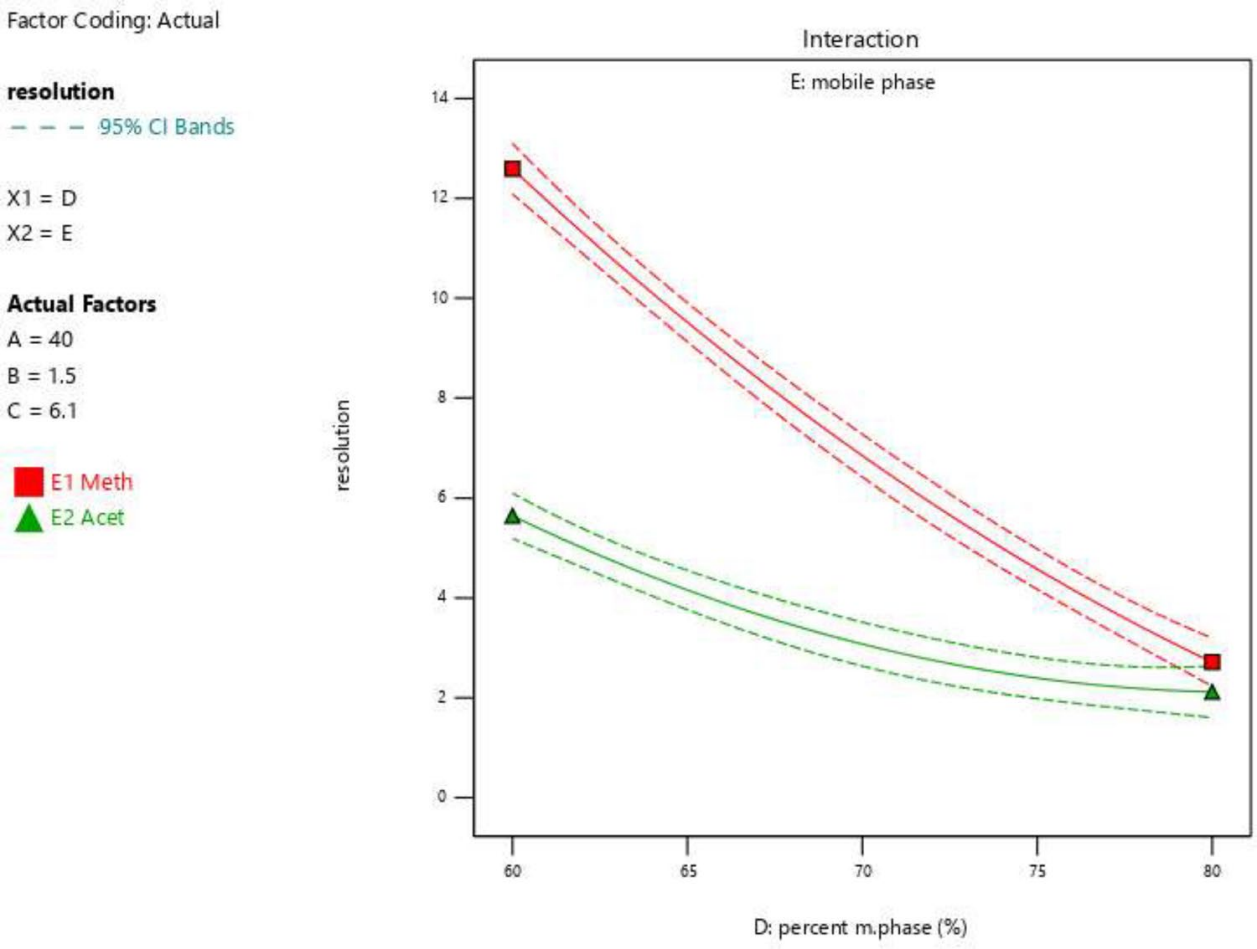
The effect of interaction of percentage of organic phase (D) and its type (E) on resolution.

The equation has been used to forecast how the variables and response relate to one another. Positive values refer to positive effect whereas an opposition interaction is indicated by negative value. For example, D and E have negative effect on resolution whereas DE, and D^2^ were discovered to be positive. It is observed from equation and ANOVA analysis that Increasing organic phase concentration (D) generally lowers resolution, but the effect is weaker when acetonitrile is used as shown in Figs. 2, 3.

**Fig. 4:**
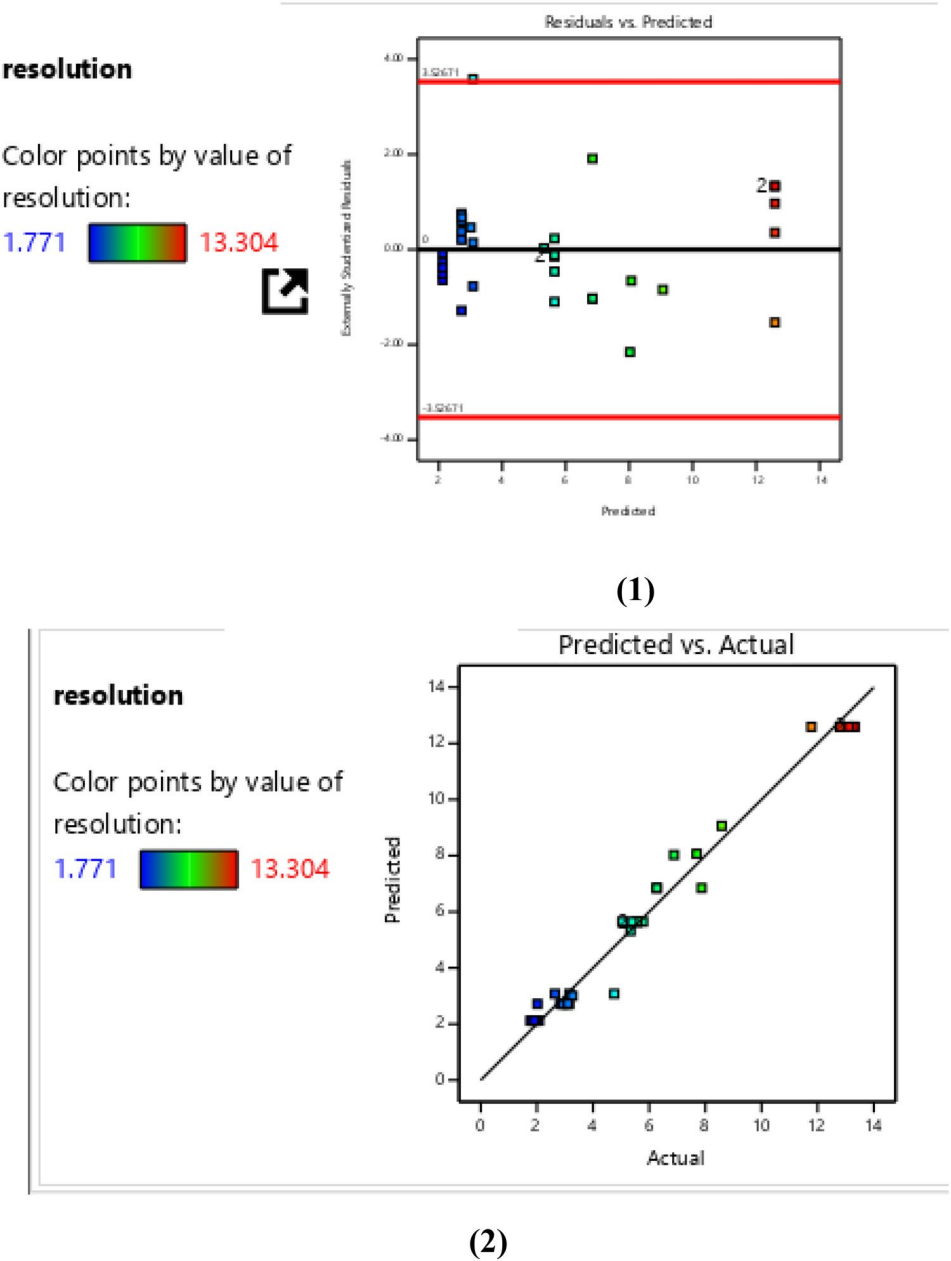
Model diagnostic plots for resolution (R_s_): (1) residual vs. predicted; (2) predicted vs. actual.

The adequacy of the quadratic regression model for resolution was evaluated using diagnostic plots (Fig. 4). The residuals vs. predicted plot (Fig. 4(1)) showed randomly scattered points around the zero line with no obvious funnel, trend, or pattern, indicating that the residuals were independent and homoscedastic (constant variance). No outlier was observed beyond ± 3.0, and the residuals were approximately normally distributed. The predicted vs. actual plot (Fig. 4(2)) demonstrated a strong linear correlation (R² = 0.975) with all points closely distributed along the diagonal line (y = x), confirming that the model predictions were in good agreement with the experimental observations. The close clustering of points around the diagonal across the entire range of resolution values (1.77 to 13.30) indicates that the model does not systematically overpredict or underpredict at low- or high-resolution values. These diagnostic checks, together with the non-significant lack-of-fit (*p* > 0.05) and high adjusted R² (0.972), confirm that the quadratic model adequately describes the relationship between the CMAs and resolution without evidence of model misspecification, heteroscedasticity, or influential outliers.

One point showed a residual of approximately 2.5, which remained within the acceptable range (± 3.0) and did not significantly influence the model, as confirmed by Cook’s distance analysis (all values < 0.5).

#### The effect of factors on retention time

To better understand the factors influencing retention time, a reduced quadratic model was employed. After eliminating non-significant terms (*p* > 0.05), the following simplified.

2 $${\text{Retention time of MP}} = {\text{ 3}}.{\mathrm{28659}} - 0.{\mathrm{983}}0{\text{54 B}} - 0.{\text{85976 D }} - 0.{\text{57181 E }} + {\text{ }}0.{\text{479386 DE }} + {\text{ }}0.{\mathrm{8}}0{\text{6549 B}}^{{\mathrm{2}}}$$

3 $${\text{Retention time of PP }} = {\text{ 4}}.{\text{51343 }} - 0.{\mathrm{7}}0{\text{6188 A}} - {\mathrm{1}}.{\text{47855 B}} - {\mathrm{2}}.{\text{6314 D}} - {\mathrm{1}}.{\text{62166 E }} + {\text{ 1}}.{\text{7299 DE }} + {\text{ 2}}.0{\text{5126 D}}^{{\mathrm{2}}}$$

The results showed that the p-value of the model for retention time response was less than 0.05 which justifies the fact that the model is significant. F-value was high and a non-significant lack of fit (*p* > 0.05) was observed. R^2^ values were 0.856 and 0.901 for MP and PP respectively. Difference between adjusted R^2^ and predicted R^2^ is less than 0.2. Indicating that the model adequately fit to the experimental results.

**Fig. 5:**
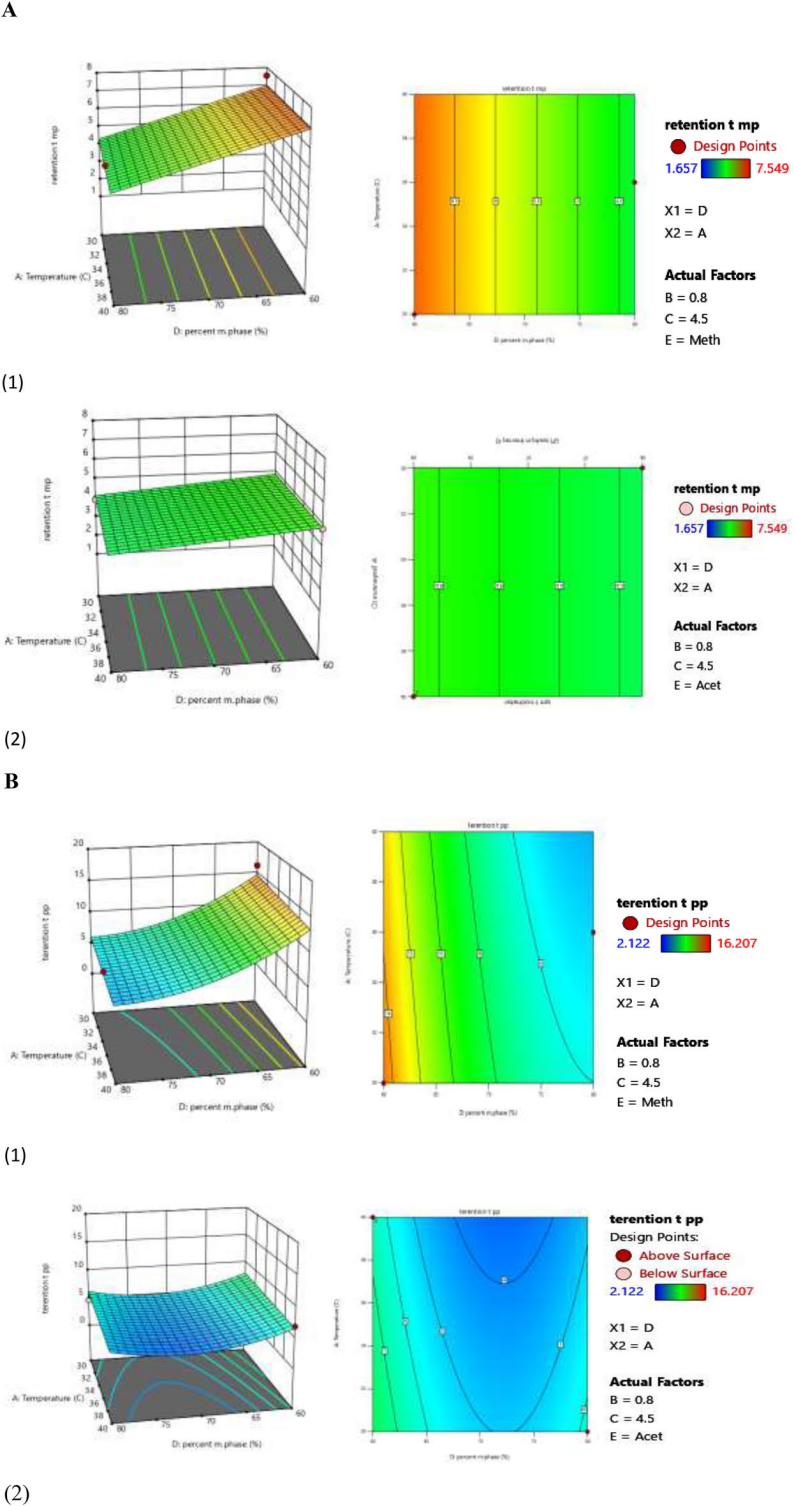
Contour plots (2D) and response surface plots (3D) showing the combined effects of organic phase percentage (X₁) and temperature (X₂) on the retention time of (**A**) methylparaben (MP) and (**B**) propylparaben (PP). The two organic solvents tested are presented separately: (1) methanol and (2) acetonitrile.

The final reduced quadratic model (Eqs. 2 and 3) identified flow rate (B), the percentage of organic phase (D), and the type of solvent (E) as the key factors influencing the retention time of methyl paraben (MP) (Figs. 5 (1), 6) The significant negative coefficients for these terms (p-value < 0.0001) indicate that increasing any of these variables reduces MP retention. This negative influence is counterbalanced by the significant positive effects of the DE interaction (*p* = 0.0008) and the quadratic flow rate term (B², *p* = 0.0019). For propyl paraben (PP), the model demonstrated a similar pattern, with retention time negatively affected by temperature (A), flow rate (B), organic phase percentage (D), and solvent type (E). The positive effect of the DE interaction was also significant for PP (Fig. 5(2), (6) along with the quadratic term for organic phase percentage (D²) (*p* = 0.0002). In both models, temperature (A) and pH (C) did not show a significant influence on MP, and pH remained non-significant for PP as well (*p* > 0.05). The greater sensitivity of propyl paraben retention to temperature, unlike methyl paraben, stems from its larger size and higher hydrophobicity. These properties promote stronger binding to the stationary phase and a larger enthalpy change (ΔH) upon transfer, making its retention time more responsive to thermal energy.

The diagnostic plots for the retention time model of methylparaben (MP) and propylparaben are presented in Figs. (7, 8). The residuals vs. predicted plot (Fig. 7(1)) showed randomly scattered points around the zero line with no obvious funnel or trend, indicating homoscedasticity and independence of residuals. Although two points exhibited residuals slightly above 1.0 (at predicted retention times of approximately 1.8 and 6.5), these remained within the acceptable range (± 3.0) and were not considered influential outliers. The predicted vs. actual plot (Fig. 7(2)) demonstrated reasonable agreement between model predictions and experimental values, with points distributed along the diagonal line. The coefficient of determination (R² = 0.856) indicates that the model explains approximately 86% of the variability in retention time, which is acceptable for a reduced quadratic model in chromatographic method development. The color-coded points (methanol vs. acetonitrile) showed no systematic clustering, confirming that the model adequately captures the effects of both mobile phase types without bias. These diagnostic checks, together with the non-significant lack-of-fit (*p* > 0.05), confirm that the reduced quadratic model appropriately describes the relationship between the CMAs and retention time for MP.

**Fig. 6:**
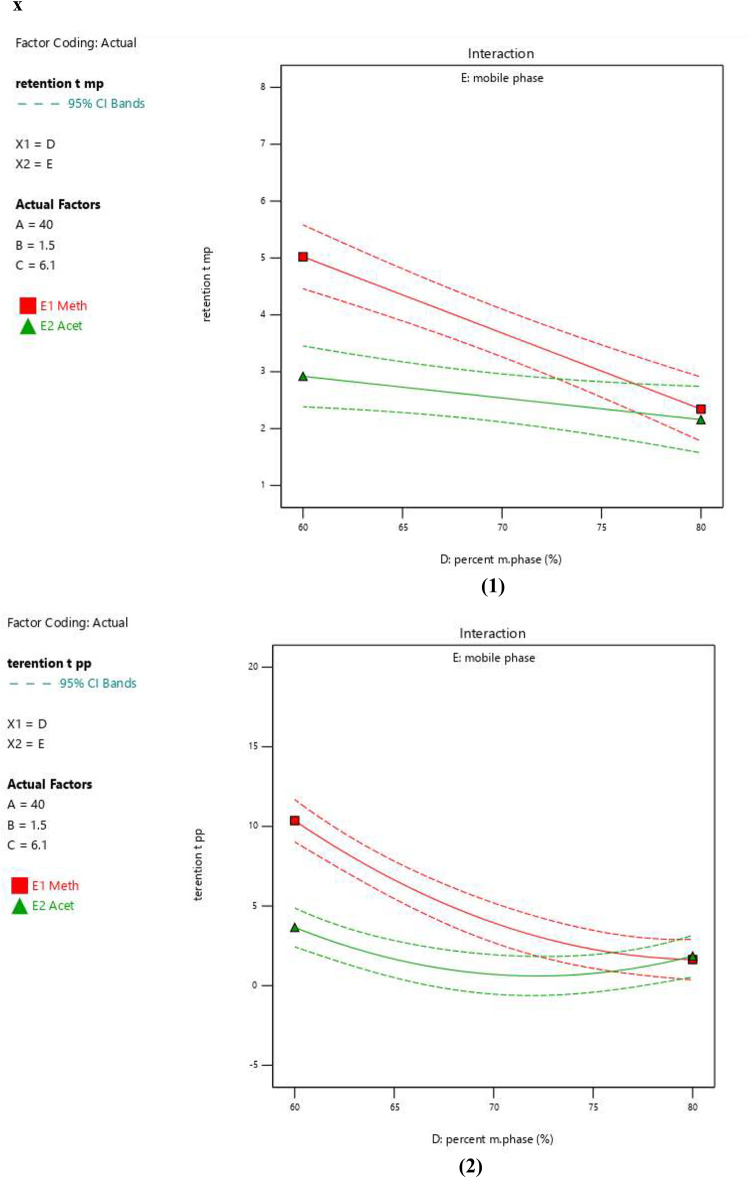
The effect of interaction of percentage of organic phase (D) and its type (E) on retention time of methylparaben (1) and propylparaben (2).

**Fig. 7:**
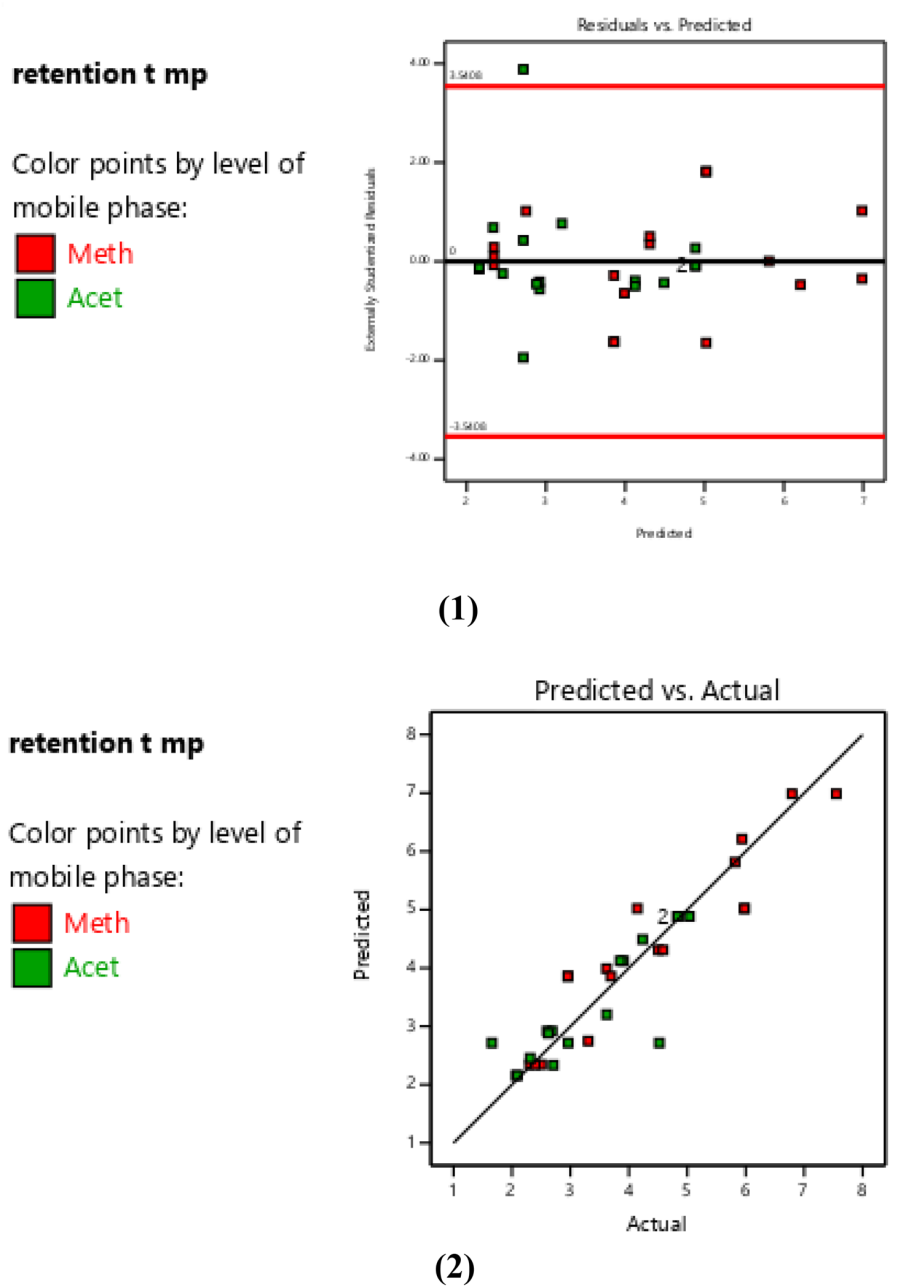
Model diagnostic plots for retention time of MP: (1) residual vs. predicted; (2) predicted vs. actual.

**Fig. 8:**
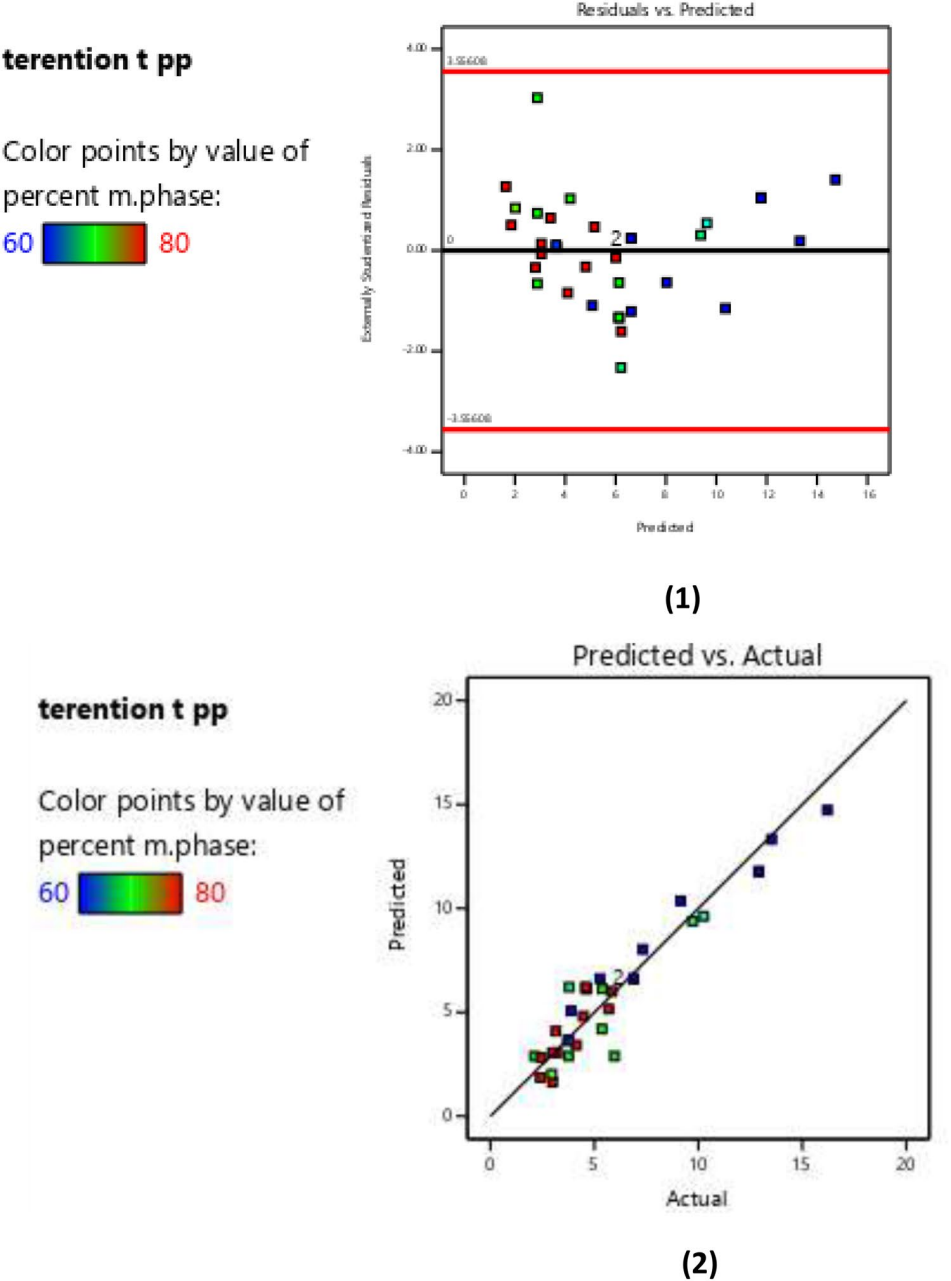
Model diagnostic plots for retention time of PP: (1) residual vs. predicted; (2) predicted vs. actual.

#### The effect of factors on tailing factor

The effect of factors on tailing factor (Tf) was analyzed using ANOVA. For both MP and PP, no significant effects of any CMA (temperature, flow rate, pH, organic phase percentage, or mobile phase type) were observed (*p* > 0.05 for all main effects and interactions). The tailing factor for MP ranged from 1.396 to 1.785, while for PP it ranged from 1.237 to 1.890. In 32 out of 34 experimental runs (94%), the Tf values were ≤ 1.5 for both analytes. Although some runs (e.g., Runs 1, 3, and 25 in Table 3) showed Tf values slightly above 1.5 (maximum 1.785 for MP and 1.890 for PP), these deviations were sporadic and not associated with any specific factor level. Residual diagnostics confirmed that ANOVA assumptions (normality and homogeneity of variance) were satisfied (Shapiro-Wilk test, *p* > 0.05; Levene’s test, *p* > 0.05). The inherently good peak symmetry can be rationalized by the chemical structure of parabens: their weak acidity (pKa ≈ 8.2–8.5) and lack of ionizable groups within the studied pH range (4.5–6.5) minimize electrostatic interactions with residual silanol groups on the C18 stationary phase. Consequently, consistent peak shapes were achieved across all experimental conditions without the need for tailing suppressors or specialized columns. Furthermore, tailing factor is not a Critical Quality Attribute (CQA) that required optimization, as it consistently met acceptance criteria across all experimental conditions without any significant factor effects (Fig. 8).

#### Experimental verification of the method operable design region (MODR)

To experimentally validate the defined MODR, nine additional verification runs were performed at the MODR boundaries, at worst-case combinations, and at random points within the design space. Table 4 presents the predicted values (from the quadratic regression models) alongside the measured values for resolution (Rs), retention time of PP (Rt), and tailing factor of MP (Tf).

The optimal conditions (Run 1: 40 °C, 1.5 mL/min, pH 6.1, 67.7% MeOH) were previously validated through system suitability testing (*n* = 6). Boundary runs (Runs 2–5) tested the four extreme corners of the MODR. Worst-case runs (Runs 6–7) tested combinations expected to produce the lowest resolution and the highest retention time. Random runs (Runs 8–9) confirmed method performance at intermediate conditions.

As shown in Table 4, all runs within the MODR boundaries (Runs 1–5, 7–9) produced acceptable CQAs with measured resolution ≥ 2.0, retention time ≤ 10 min, and tailing factor ≤ 1.5. At the extreme upper boundary combination (Run 6: 42 °C, 1.6 mL/min, pH 5.9, 70% MeOH), the measured resolution was 1.99, slightly below the acceptance criterion of 2.0. This confirms that the MODR limits (65–70% MeOH, pH 5.9–6.3, flow rate 1.4–1.6 mL/min, temperature 38–42 °C) are appropriately defined, and operating outside these limits may compromise method performance.

The close agreement between predicted and measured values (differences ≤ 0.03 for Rs, ≤ 0.04 min for Rt, and ≤ 0.02 for Tf) confirms the good predictive capability of the quadratic regression models.

Based on these results, the MODR is considered experimentally validated. The recommended operating conditions for routine quality control are the optimal point (40 °C, 1.5 mL/min, pH 6.1, 67.7% MeOH), with allowable variations within the MODR limits as defined above. According to ICH Q14 guidelines, this experimentally verified MODR serves as evidence of method robustness without requiring separate multi-instrument or multi-analyst testing.

**Table 4:** Experimental verification of MODR boundaries – predicted vs. measured values for resolution (Rs), retention time (Rt), and tailing factor (Tf). Acceptance criteria: Rs ≥ 2.0, Rt ≤ 10 min, Tf ≤ 1.5.

Temperature (C˚)	Flow rate (ml/min)	pH	Methanol (%)	Predicted (*R*_S_)	Measured (*R*_S_)	Predicted (*R*_t)_	Measured (*R*_t)_	Predicted (Tf)	Measured (T_f)_
40	1.5	6.1	67.7	3.2	3.22	5.8	5.81	1.13	1.13
38	1.4	5.9	65.0	2.5	2.49	6.5	6.53	1.18	1.17
42	1.6	6.3	70.0	2.2	2.18	4.8	4.76	1.20	1.21
38	1.6	5.9	70.0	2.4	2.36	5.2	5.18	1.17	1.18
42	1.4	6.3	65.0	2.1	2.09	6.0	6.04	1.21	1.20
42	1.6	5.9	70.0	2.0	1.99	5.0	4.95	1.22	1.23
38	1.4	6.3	65.0	2.3	2.25	6.8	6.82	1.19	1.19
39	1.45	6.0	68.5	2.8	2.83	5.4	5.43	1.1	1.14
41	1.55	6.2	66.5	2.6	2.62	5.6	5.62	1.1	1.13

#### Integration of FMEA with DoE results

The quantitative FMEA performed during the risk assessment phase (Table 1) successfully identified the most critical CMAs, and the DoE results largely confirmed these predictions. Table 5 summarizes the comparison between FMEA risk rankings and DoE outcomes.

**Table 5:** Validation of FMEA predictions against DoE outcomes.

Parameter	RPN	DoE outcome (*p*-value for *R*_s_)	Agreement with FEMEA
Mobile phase composition	240	< 0.0001	Yes
Mobile phase pH	240	0.008	Yes
Flow rate	192	0.03	Yes
Temperature	140	0.08	Overestimated

Specifically, mobile phase composition (RPN = 240) was confirmed as the most influential factor, showing significant effects on resolution (*p* < 0.0001), retention time (*p* < 0.0001), and tailing factor (*p* = 0.002). Mobile phase type (methanol vs. acetonitrile) also significantly affected resolution (*p* = 0.001), with methanol providing superior performance. Mobile phase pH (RPN = 240) showed a significant quadratic effect on resolution (*p* = 0.008) and a linear effect on retention time (*p* = 0.01), confirming its high-risk classification. Flow rate (RPN = 192) significantly reduced retention time (*p* < 0.0001) and showed a minor effect on resolution (*p* = 0.03), consistent with FMEA predictions. Temperature (RPN = 140) significantly affected retention time (*p* = 0.02) but showed no significant effect on resolution (*p* = 0.08), suggesting that the FMEA overestimated its impact on resolution within the studied range (30–40 °C). Medium-risk factors (wavelength, injection volume, column type, sample dilution) were excluded from the DoE with justifications based on literature, preliminary experiments, or practical considerations, as detailed in the Experimental section. This integration of FMEA and DoE demonstrates that the risk assessment was not merely procedural but accurately guided the experimental design and interpretation of results.

#### Optimized condition obtained

Following the identification of significant factors, an optimization of the chromatographic conditions for maximum resolution and minimum analysis time was employed. The optimal conditions were predicted to be 40 °C temperature, 1.5 mL/min flow rate, pH 6.1, with 67.7% methanol as the organic phase. Although the dominance of organic solvent percentage is expected, the systematic quantification of its interaction with pH and temperature provides added value. Methanol was selected over acetonitrile based on the MODR analysis, as acetonitrile resulted in lower resolution and higher tailing. Detailed thermodynamic or molecular-level explanation of solvent effects is beyond the scope of this method development study. Under these conditions, high desirability score of 0.638 which represented the best practical compromise between maximizing resolution (> 5) and minimizing total analysis time (< 6 min) within the MODR. A desirability of 0.638 is considered acceptable for method development purposes when multiple CQAs are simultaneously optimized.

### Method validation

#### System suitability

**Fig. 9:**
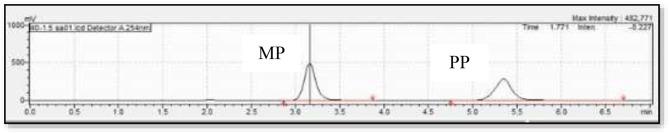
Chromatogram showing methylparaben (MP) and propylparaben (PP) under optimized condition.

The system chromatographic reproducibility for 6 injections was checked. Retention time, tailing factor and theoretical plate were obtained. The value of standard deviation (SD) and relative standard deviation (%RSD) indicate the reproducibility of the chromatographic system is good (Table 6; Fig. 9) All values of %RSD were in acceptance criteria (below 2%). All values for tailing factors were below 1.5 and for theoretical plates were over 2000. Peak purity was not assessed due to instrument limitations (single-wavelength UV detector). precision acceptable without internal standard, matrix effects minimal, cost and simplicity advantages.

**Table 6:** System suitability test results (*n* = 6) under optimized chromatographic conditions.

	Retention time		Tailing factor		Theoretical plates	
	MP	PP	MP	PP	MP	PP
1	3.192	5.347	1.126	0.693	3045	4540
2	3.190	5.348	1.127	0.692	3040	4520
3	3.196	5.347	1.129	0.694	3027	4550
4	3.192	5.346	1.125	0.685	2053	4530
5	3.195	5.349	1.127	0.693	3045	4560
6	3.193	5.348	1.128	0.695	3019	4540
Average	3.193	5.347	1.127	0.692	3035	4540
SD	0.002	0.001	0.004	0.003	11.14	14.14
%RSD	0.07	0.02	0.13	0.48	0.37	0.31

*SD* standard deviation (*n* = 6), *%RSD* relative standard deviation.

#### Linearity

**Fig. 10:**
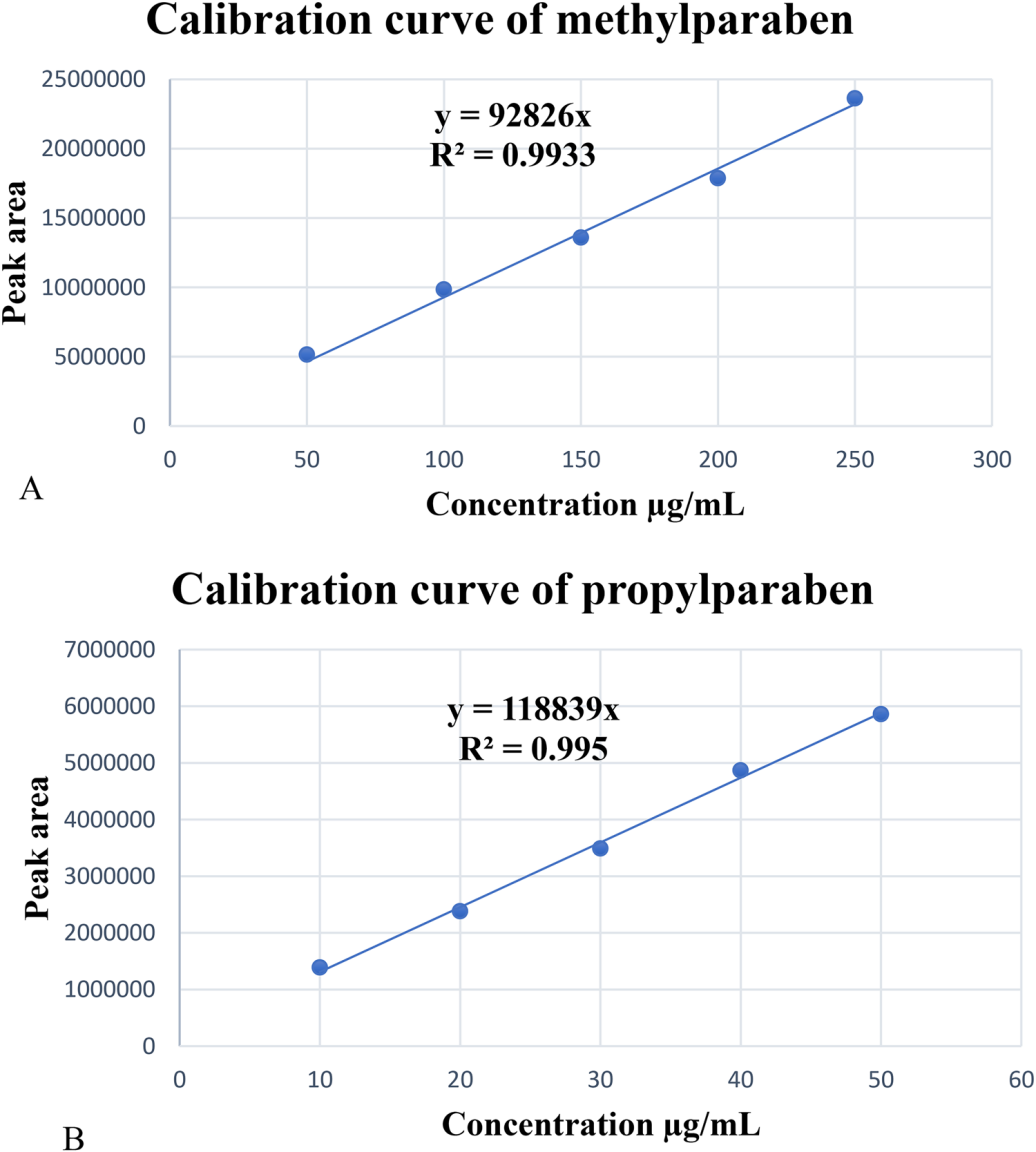
Calibration curve of methylparaben (**A**) and propylparaben (**B**).

The calibration curve of standard solution of MP is (50–250 µg/ml) and (10–50 µg/ml) for PP was plotted in between average peak ration and concentration. The results are shown in Fig. 10. The R^2^ value 0.993 and 0.995 for MP and PP respectively indicated good linearity. Five calibration levels are acceptable for linearity assessment in routine quality control according to ICH Q2(R2).

### Precision and accuracy

Inter and intraday precision are presented in Table 7. %RSD of the precision and accuracy was below 2% (Table 8), which indicate that the developed method is precise and accurate. Intermediate precision was evaluated over only two days; three or more days would provide more robust estimates.

**Table 7:** Precision.

	Inter-day			Intraday		
	Theoretical concentration (µg/ml)	Concentration found (Mean ± SD)	%RSD	Theoretical concentration (µg/ml)	Concentration found	%RSD
MP	80	79.56 ± 0.3	0.38	80	79.92 ± 0.41	0.51
	100	100.5 ± 0.03	0.03	100	100.5 ± 0.05	0.05
	120	118.93 ± 0.6	0.50	120	116.65 ± 0.7	0.60
PP	16	16.40 ± 0.02	0.12	16	16.21 ± 0.06	0.37
	20	19.76 ± 0.39	1.97	20	19.82 ± 0.33	1.67
	24	23.78 ± 0.47	1.98	24	23.67 ± 0.35	1.48

**Table 8:** Accuracy.

Level of added concentration (%)	Theoretical amount (µg)	amount found (µg) (Mean ± SD)	%Recovery (Mean ± SD)	%RSD
50	150	148.3 ± 0.2	98.87 ± 0.13	0.13
100	200	198.3 ± 0.02	99.15 ± 0.01	0.01
150	250	249.5 ± 0.3	99.80 ± 0.12	0.12
50	30	29.70 ± 0.2	99.0 ± 0.67	0.67
100	40	39.92 ± 0.1	99.80 ± 0.25	0.25
150	50	49.12 ± 0.04	98.24 ± 0.08	0.08

#### Robustness

In contrast to traditional one-factor-at-a-time (OFAT) robustness testing (which requires separate experiments on different instruments, columns, or analysts), the AQbD paradigm establishes robustness through the definition and experimental verification of the Method Operable Design Region (MODR). Within the MODR, all Critical Quality Attributes (CQAs) are guaranteed to meet acceptance criteria despite deliberate variations in Critical Method Attributes (CMAs).

The MODR boundaries (MeOH 65–70%, pH 5.9–6.3, flow rate 1.4–1.6 mL/min, temperature 38–42 °C) were experimentally challenged at their extreme corners and at worst-case combinations (highest temperature + highest flow rate + lowest pH, Table 3). At all tested points within the MODR, resolution (Rs) remained ≥ 2.0, retention time ≤ 10 min, and tailing factor ≤ 1.5. At a deliberately chosen point outside the MODR (42 °C, 1.6 mL/min, pH 5.9, 70% MeOH), resolution dropped to 1.99, confirming the boundaries are correctly defined. This experimental verification serves as evidence of robustness without requiring separate multi-instrument or multi-analyst testing, as per ICH Q14 guidelines.

#### Specificity

Specificity was confirmed by comparing chromatograms of standard solutions, sample solutions, and a placebo solution containing common syrup excipients (sucrose, glycerin, flavors, colorants). No interfering peaks were observed at the retention times of MP (approximately 3.2 min) and PP (approximately 5.8 min). To further evaluate potential matrix effects, the matrix factor was calculated by comparing the slope of the calibration curve prepared in mobile phase with that prepared in placebo matrix. The matrix factor (slope ratio) was 1.01 for MP and 0.99 for PP, confirming negligible matrix interference.

#### Limit of detection (LOD) and limit of quantification (LOQ)

LOD and LOQ were calculated from the calibration curves using the standard deviation of the intercept (σ) and the mean slope (S) according to the formulas: LOD = 3.3 × σ/S and LOQ = 10 × σ/S. As presented in Table 9, the LOD values were 0.15 µg/mL for MP and 0.08 µg/mL for PP, while the LOQ values were 0.50 µg/mL for MP and 0.27 µg/mL for PP. These low values demonstrate the high sensitivity of the developed method, allowing accurate detection and quantification at trace levels. LC-MS would offer higher sensitivity, but the current LOD (0.15 µg/mL) is adequate for syrup QC where working concentrations are 50–250 µg/mL for MP and 10–50 µg/mL for PP.

The signal-to-noise ratio (S/N) was estimated using the peak-to-peak noise method. At the working concentration (MP 100 µg/mL, PP 20 µg/mL), the S/N was approximately 2000:1 for MP and 3700:1 for PP. At the LOQ concentration, the S/N was 10:1 for both analytes, confirming the sensitivity of the method according to ICH Q2(R2) guidelines.

**Table 9:** Limits of Detection (LOD) and Quantification (LOQ) for Propylparaben and Methylparaben Using the HPLC Method.

Paraben compound	LOD (µg/mL)	LOQ (µg/mL)	LOD at S/*N*	LOQ at S/*N*	S/*N*
MP	0.15	0.50	3:1	10:1	~ 2000:1 (100 µg/ml)
PP	0.08	0.27	3:1	10:1	~ 3700:1 (70 µg/ml)

The developed method is intended for routine quality control quantification of MP and PP in pharmaceutical syrups within their labeled shelf life. No claim is made regarding stability-indicating capability, as forced degradation studies according to ICH Q1A were not performed. For applications requiring stability-indicating methods (e.g., long-term stability studies or analysis of degraded products), additional validation including forced degradation would be necessary. This limitation has been acknowledged in the Conclusion section.

#### Application to the real samples

The validated method was applied to the determination of MP and PP in pharmaceutical syrups. The samples were analyzed without prior knowledge of their paraben content. Each sample was prepared in triplicate injected under the optimized chromatographic conditions. The concentrations of MP and PP in the analyzed samples are presented in Table 10. The results showed that MP concentrations ranging from 0.09 to 0.15% and PP concentrations ranging from 0.01 to 0.02%.These values fall within the typical range for parabens used as preservatives in pharmaceutical syrups.

**Table 10:** Methylparaben and propylparaben concentration in pharmaceutical syrups.

Sample	Analyte	Concentration in samples (µg/ml)	%RSD	Concentration in pharmaceutical syrups (w/v)%
1	MP	120 ± 0.2	0.17	0.12
	PP	10 ± 0.05	0.5	0.01
2	MP	150 ± 0.03	0.02	0.15
	PP	20 ± 0.2	1	0.02
3	MP	90 ± 0.3	0.33	0.09
	PP	10 ± 0.01	0.1	0.01

#### 10-fold dilution factor

The calibration ranges (50–250 µg/mL for MP and 10–50 µg/mL for PP) were selected based on the expected paraben concentrations in pharmaceutical syrups. Based on literature and preliminary analysis, MP concentrations in undiluted syrups typically range from 0.09 to 0.15% w/v (900–1500 µg/mL) and PP from 0.01 to 0.02% w/v (100–200 µg/mL). Following the 1:10 sample dilution procedure described in Sect. 2.4, the expected concentrations in the injected solution are 90–150 µg/mL for MP and 10–20 µg/mL for PP, which fall well within the selected calibration ranges.

#### Comparison with previously reported HPLC methods

To assess the relative performance of the developed AQbD-assisted method, a comparison was made with three previously reported HPLC methods for simultaneous determination of MP and PP in pharmaceutical formulations (Table 11). Our method offers comparable or superior performance in several aspects, most notably the explicit definition of a Method Operable Design Region (MODR), which most conventional methods lack. The comparison shows that our 6-minute run time is within the typical range for conventional HPLC analysis of parabens (5–12 min). Attempts to further reduce the run time (e.g., by increasing flow rate > 1.5 mL/min or organic solvent > 70%) resulted in a significant loss of resolution (Rs < 2.0) and increased backpressure (> 250 bar), which is not advisable for routine QC. Therefore, the selected conditions represent a practical optimum balancing speed, resolution, and column longevity. The combination of a defined design space, systematic DoE optimization, and validated robustness within the MODR represents a methodological advancement over traditional one-factor-at-a-time approaches. As the goal was not superiority but systematic AQbD application, the validation results meet ICH Q2(R2) acceptance criteria and are adequate for routine quality control purposes.

**Table 11:** Comparison of the developed method with published HPLC methods for MP and PP determination.

Parameter	Our method	Ref 1 (2025)^25^	Ref 2 (2023)^26^	Ref 3(2013)^27^
Column	C18 (150*4 mm, 5 μm)	C18 (250*4.6 mm, 5 μm)	C18 (250*4.6 mm, 5 μm)	C18 (250*4.6 mm, 5 μm)
Run time (min)	6	30	7	6
LOD MP and PP (µg/mL)	0.15	0.37 and 2.002	1 and 1	1.50 and 0.50
AQbD	Yes	No	No	No

## Limitations and future directions

While this study successfully demonstrates the application of AQbD principles to define and experimentally verify a MODR for the simultaneous determination of MP and PP, several limitations should be acknowledged.

This method was developed and validated solely for routine quality control quantification of MP and PP within the labelled shelf life of the products. No claim is made regarding stability-indicating properties, and forced degradation studies according to ICH Q1A were not performed. For stability studies, additional validation would be required.

An internal standard (e.g., ethyl paraben) was not used. The method achieved acceptable precision (%RSD < 1.5%) and accuracy (98–102%) without it, as permitted by ICH Q2(R2) when method performance is adequate. However, for highly complex or unknown matrices, an internal standard is recommended.

Due to the use of a single-wavelength UV detector (without PDA or MS), peak purity was not assessed. While no interfering peaks were observed in the tested commercial syrups, co-elution with unexpected excipients or degradation products cannot be completely excluded.

According to AQbD principles, robustness is demonstrated by establishing and experimentally verifying the Method Operable Design Region (MODR), not by exhaustive multi-instrument testing. The MODR (MeOH 65–70%, pH 5.9–6.3, flow rate 1.4–1.6 mL/min, temperature 38–42 °C) was experimentally verified at its boundaries and at worst-case combinations (Table 3). Transfer to other laboratories is facilitated by the defined MODR, not by post-hoc testing. Full lifecycle management (ICH Q14) is beyond the scope of this single-study manuscript.

Formal uncertainty estimation according to EURACHEM/CITAC guidelines was not calculated and is recommended if the method is to be used for accredited regulatory purposes.

Addressing the remaining aspects would further strengthen the AQbD framework presented here and facilitate the transition of this method from research to routine quality control use.

## Conclusion

A robust HPLC method for the simultaneous determination of methylparaben (MP) and propylparaben (PP) in liquid oral dosage forms was successfully developed using a systematic AQbD approach. Risk assessment (Ishikawa diagram and FMEA) identified five critical method attributes (CMAs): mobile phase type, organic phase percentage, pH, flow rate, and temperature. These were optimized using a D-optimal design (34 runs). The systematic optimization not only reduced method development time but also provided a deep understanding of factor interactions, resulting in a method with a well-defined design space.

Statistical analysis revealed that organic solvent type and percentage were the most significant factors affecting resolution and retention time (*p* < 0.0001). Mobile phase pH showed a significant quadratic effect on resolution (*p* = 0.008), confirming its high-risk classification in the FMEA. Flow rate significantly reduced retention time (*p* < 0.0001), while temperature had a larger effect on propylparaben than methylparaben (*p* = 0.02). Tailing factor remained unaffected across all conditions.

The Method Operable Design Region (MODR) was established, with optimal conditions of 40 °C column temperature, 1.5 mL/min flow rate, mobile phase pH 6.1, and 67.7% methanol. Under these conditions, baseline separation (Rs = 3.2) was achieved within 6 min (retention time of PP = 5.8 min). The overall desirability of the optimized conditions was 0.638, which is considered acceptable for multi-response optimization balancing resolution, retention time, and tailing factor.

Based on the established MODR, a proposed control strategy including system suitability test acceptance criteria (Rs ≥ 2.0, Tf ≤ 1.5, %RSD ≤ 2.0%) and control limits for critical parameters (MeOH 65–70%, pH 5.9–6.3, flow rate 1.4–1.6 mL/min, temperature 38–42 °C) has been defined to facilitate routine application.

The method was validated according to ICH Q2(R2) guidelines, demonstrating that the developed method can be adapted for the routine quality control of parabens in pharmaceutical syrups. The MODR boundaries were experimentally verified through independent validation runs at the limits of the design space and at worst-case combinations (Table 4), confirming that all CQAs remain within acceptance criteria when operating within the defined ranges.

## Data Availability

The data presented in this study are available on request from the corresponding author.
